# Hip Mobilization at Preterm Age May Accelerate Developmental Dysplasia Recovery

**DOI:** 10.1155/2018/8625721

**Published:** 2018-10-29

**Authors:** Mariana Callil Voos, Soares de Moura Maria Clara Drummond, Renata Hydee Hasue

**Affiliations:** Department of Physical Therapy, Communication Sciences and Disorders and Occupational Therapy, Faculty of Medicine, University of São Paulo, Brazil

## Abstract

**Purpose:**

Few studies have described mobilization approaches in developmental dysplasia of the hip (DDH). The present study describes the hip mobilization of a preterm infant (born at 33 6/7 weeks of gestational age) diagnosed with DDH.

**Design and Methods:**

During the 43-day hospital stay, the infant was seen twice a week (ten sessions, 20 minutes each). All sessions included hip approximation maneuvers, with the hip positioned in abduction, lateral rotation and flexion, and lower limbs passive mobilization, which were taught to the mother. Early intervention with auditory, tactile, visual, and vestibular stimulations was also performed. The infant was assessed with hip ultrasound before and after treatment.

**Results:**

At 34 2/7 weeks of gestational age, she was classified as Graf IIa (left: alpha: 55°, beta: 68°; right: alpha: 59°, beta: 64°). At 40 5/7 weeks, she was classified as Graf I for left (alpha: 67°; beta: 42°) and right (alpha: 66°; beta: 42°) hips.

**Practical Implications:**

The intervention seemed to accelerate the acquisition of stability of dysplasic hips in a preterm infant. The outcome supports further investigation of hip approximation maneuvers as part of early stimulation in preterm infants with DDH during hospital stay.

## 1. Introduction

Developmental dysplasia of the hip (DDH) ranges from temporary hip instability to total hip dislocation [1]. Abnormal hip development may involve bone structures (acetabulum and proximal femur) and the joint cartilage and capsule [2, 3]. The incidence varies from 1.5 to 25.0 per 1,000 live births, depending on diagnostic criteria, population, and screening method [1, 2]. Risk factors include breech position, female sex, twin pregnancy, positive family history, white race, primiparous young mother, oligohydramnios, macrosomy, and foot, knee, or spine deformities [2, 4]. However, 75% of DDH cases occur in female infants without any other risk factors [1, 2]. DDH is more common in the left hip [2]. The left hip is more frequently affected than the right one because, in cephalic presentation, most fetuses have their spines positioned to the left of the mother. This position may cause restrained abduction of the fetus left hip [5].

Few studies have investigated DDH in preterm infants. Quan et al. [6] studied the incidence of DDH in infants born in breech position. They concluded that preterm and term infants had a similar incidence of DDH and, therefore, required similar screening approaches. Chan et al. [4] reported that preterm infants showed a lower risk of DDH than terms. However, a more recent study, by Sezer et al. [7], concluded that prematurity did not influence DDH prevalence. DDH may have severe consequences if not diagnosed and treated in a timely manner [8]. The main consequences are arthritis and pain, which may lead to childhood disability, such as hip instability at walking age, gait impairment and/or delay, and even the need for joint arthroplasty [1, 9].

Hip examination is part of the first evaluation of the newborn. Abnormal clinical examination (positive Barlow and/or Ortolani tests, limited abduction, and asymmetrical skin folds) determines the need of further investigation [10]. A coronal sonogram must be obtained with the infant positioned in lateral decubitus. The hips must be flexed, adducted, and medially rotated. The Graf method can classify DDH severity [11–13]. Two angles are formed by three lines drawn from the acetabular lateral edge, bottom, and labrum. The bony roof angle is known as the alpha angle, and the cartilage roof angle is known as the beta angle.

Type Ia and Ib Graf classifications mean mature hips, with alpha angle higher than 60° (type I). Type IIa means immature hips (hips that can subluxate/dislocate, although reduced in the acetabulum), with alpha angle higher than 50° (in infants younger than three months). Types IIb and IIc denote dysplasic hips (femoral head in the correct position, but with partial contact with the acetabulum), with alpha angle lower than 50° [2, 14, 15]. Infants classified as IIa show risk of hip dislocation because hips may become decentralized [16] (Omeroglu et al., [17]). Although most hips classified as IIa show significant spontaneous clinical improvement, some hips can become IIb or even more unstable by the age of seven weeks (Omeroglu et al., [17]). Therefore, all infants whose hips are classified as IIa must be followed, regardless of gestational age at birth or at instability detection [16]. According to Omeroglu et al. [17]), IIa hips are more common in newborn girls, and they are less likely to spontaneously normalize in girls than in boys. The authors state that many parents do not receive enough explanations regarding the type IIa hip risks and miss follow-up appointments (Omeroglu et al., [17]).

Early intervention can provide a better outcome [1, 18]. The treatment for children younger than six months is splinting, mainly by the Pavlik harness [19–22], as well as for infants classified as II or higher (Atalar et al., [23]). Hip flexion and abduction and knee flexion maintain hip motion and result in the gentle reduction of the hip [21, 24]. The reported duration of splinting varied from 11 to 32 weeks [21].

However, hip mobilization can reduce instability and minimize the risk of hips classified as IIa. Specific maneuvers may accelerate and assure a positive outcome, without causing any movement restriction. As IIa hips are considered as having mild instability, the restriction provided by Pavlik harness may not be a good clinical option, when cost-benefit (movement restriction versus hip stability) is considered. Besides, the regular observation of spontaneous movements in preterm and term infants is important to follow up their development [25].

Very few studies show positive outcomes with infants younger than two months. Preterm babies are more likely to have immature hips, and DDH can be detected in the first days after birth. However, no study has proposed any specific intervention for this population. Therapeutic mobilization techniques have not been tested specifically in DDH but have been shown to improve joint stability. Proprioceptive neuromuscular facilitation (PNF) improved joint stabilization in young adults [26]. PNF employs joint approximation to stimulate joint receptors, facilitate alpha motor neurons, and promote reflex muscle contraction in order to improve joint stability [27]. We hypothesized that an early treatment protocol, which also considered the best hip positioning (abduction, flexion, and external rotation and joint approximation) and stimuli to provide better hip positioning and stability, could be helpful to treat a premature newborn patient with DDH.

### 1.1. Case Report

Patient MEFR, a preterm infant, born at 33 6/7 weeks, stayed in University Hospital for 43 days and was discharged at 39 6/7 weeks. Her mother, JFR, was primiparous, 31 years old, and adequately followed up during gestation. The premature delivery was caused by chorioamnionitis and preterm placental dystrophic calcification.

When placental calcification occurs before 36 weeks of gestation, it is preterm placental dystrophic calcification [28]. Preterm placental dystrophic calcification is associated with risks for adverse maternal outcomes (hemorrhage, placental abruption, and transfer to the obstetric intensive care unit) and for adverse fetal outcomes (preterm delivery, low weight, low Apgar score, and even death) [28]. MEFR was born in cephalic presentation, by vaginal delivery, and received the Apgar score of 8/9/10. Her Capurro gestational age was 33 6/7 weeks. The infant's weight was 1765 g (3.89 lb) at birth, and she was 40 cm (15.75 in) tall. Barlow and Ortolani maneuvers were positive for the left lower limb at the first clinical examination on the day of birth. She showed no spasticity or flaccid paralysis.

Hip ultrasound at 34 2/7 weeks showed noncalcified femur epiphysis, centered on the acetabular cavity, round promontory, and triangular cartilaginous tissue in the anterior region, classified as Graf IIa (alpha: 55°; beta: 68°) for the left limb and Graf Ib (alpha: 60°; beta: 64°) for the right limb.

The infant was assisted with CPAP in ICU for four hours, due to precocious respiratory discomfort. On her second day of life, she showed episodes of apnea and was treated with caffeine for 10 days. During this period, jaundice was also observed, and she was treated with phototherapy. Gastroesophageal reflux was diagnosed and treated with ranitidine. She was regularly breastfed since her second day of life. BERA, EEG, and transfontanelle ultrasound were performed on the third day of life and repeated before discharge and classified as normal. Echocardiography showed patent oval foramen and pulmonary artery stenosis.

**Figure 1:**
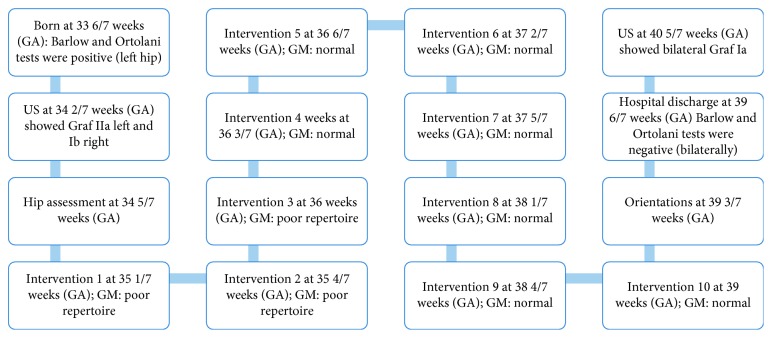
Timeline showing the main events during hospital stay. GA: gestational age.

Before all sessions, spontaneous general movements [29] were assessed. Spontaneous movements were classified as poor repertoire in the first three sessions and as normal in the subsequent seven sessions. In preterm and term infants, spontaneous general movements are classified as normal when variability and fluidity can be observed. When movements lack one or more of these characteristics, they are classified as poor repertoire [25]. She was assessed by a therapist when she was 34 5/7 weeks and treated with ten sessions of mobilization and early intervention. When she was 39 3/7 weeks, her mother received additional orientations for global stimulation prior to hospital discharge, which are given to all preterm babies, in order to prevent developmental delay (Figure 1).

The patient was monitored during the sessions and maintained adequate respiratory (42–60 breaths/min) and cardiac (144–179) frequencies and oxygen saturation (95–99%). Sessions were performed when the infant was on a quiet or active alert state.

**Figure 2:**
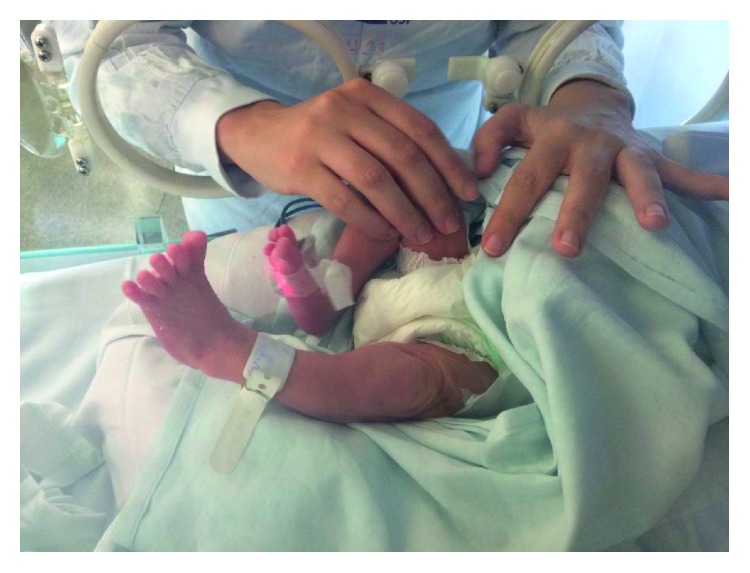
Hip mobilization.

All sessions included both hip joints' approximation maneuvers, with the infant in supine position and the hip positioned in abduction, lateral rotation and flexion, and passive mobilization of hips, knees, and ankles for about ten minutes (five minutes each leg). Joint approximation is a rehabilitation technique whereby joint surfaces are pressed together. It is used to facilitate co-contraction of muscles around a joint and thus to increase joint instability. In a preterm infant, if the femur and the pelvis are not aligned correctly, or if too much force is applied, hip luxation may occur. A trained therapist performed the maneuvers (Figure 2).

The hip joint approximation maneuver consisted of compressing through the long axis of the femur, making sure that the joint was in the correct alignment prior to approximating the femur head and the accetabulum. The approximation was held by the therapist for about one second, released for about two seconds, and repeated. After ten repetitions, a 10-to-15-second rest was given, and a new series was started. Then, the therapist performed the same series on the other hip. Four series, ten repetitions each, were performed on each side. The therapist applied only the force enough to move the limb with one hand and stabilized the hip with the other hand (Figure 2).

The mother was instructed not to perform hips adduction, extension, and medial rotation during any manipulation. She practiced the hip approximation maneuver on the first and second sessions and was instructed and observed by the therapist. On these two initial sessions, the mother was asked to place her hands on the therapist's hands, during the maneuver. The therapist performed about ten to fifteen repetitions on each hip with the mother's hands on her hands to demonstrate the correct pressure and positioning. After that, the mother performed ten to fifteen more repetitions, and the therapist observed carefully and provided feedback whenever necessary.

On the third session, the mother was instructed to perform the same movements four times a day for ten minutes (same series than during the sessions). On the days of therapy sessions, the mother performed three extra series instead of four.

As hips were initially classified as IIa, they displayed incorrect acetabulum centralization but had a low risk of subluxation, mainly when positioned externally rotated with abduction and flexion. For this reason, the mother was allowed to perform the maneuvers after two days of training. During sessions 3 to 10, the mother was asked by the therapist to show how she was performing the maneuvers, and the demonstrations were correct. The therapist also asked if she was performing the maneuver with the proposed frequency (three/four times a day), and she told that it was possible to keep that amount of daily repetitions.

During all sessions, after performing the maneuvers on the hips, the therapist performed auditory, tactile, visual, and vestibular stimulations to facilitate behavioral organization, for about ten minutes, helping the infant achieve alert states [30]. Auditory (talking), tactile (massage), and visual (eye to eye and showing toys) stimulations were performed for eight minutes, followed by two minutes of vestibular stimulation (prone and supine horizontal rocking in anteroposterior and laterolateral directions) [30, 31].

She was discharged when she was 39 6/7 weeks and had negative Barlow and Ortolani tests. Her discharge weight and height were 2770 g (6.11 lb) and 46 cm (18.11 in). When she was 40 5/7 weeks, hip ultrasound showed noncalcified femur epiphysis, centered on the acetabular cavity, round promontorius, and triangular cartilaginous tissue in the anterior region, classified as Graf I for the left (alpha: 67°; beta: 42°) and right (alpha: 66°; beta: 42°) hips.

## 2. Discussion

DDH may take many years to resolve. The present study shows relevant evidence that mobilization may accelerate the recovery of DDH, even in infants before the term age. The patient was followed up and treated with ten sessions of mobilization and early intervention during 43 days of hospital stay and was discharged with normal hip angles, at term age.

As in most cases from the literature, in the present case study, DDH affected the left hip more seriously than the right hip and the patient was female [1, 2, 4, 5]. Although preterm infants seem to have a lower risk of DDH than terms [4], clinical hip examination revealed abnormal responses on the left hip (Barlow and Ortolani maneuvers were positive for the left hip). Ultrasound examination classified the left hip as Graf IIa (alpha: 55°; beta: 68°) and the right as Ib (alpha: 60°; beta: 64°). Type IIa meant immature hips (positioned in the acetabulum but likely to subluxate).

Although gold-standard treatment for children younger than six months is abduction splints [19–22], with hip flexion and abduction and knee flexion, the brace is usually provided at hospital discharge. Therefore, we treated the patient with an experimental protocol during hospital stay, in order to optimize hip stability in this period and to avoid the use of Pavlik harness at term age.

The literature-reported duration of treatment in the Pavlik harness varied from 11 to 32 weeks [21]. In the present study, the left hip was classified as normal after 43 days (6 weeks) of hospital stay. In this period, the patient underwent 10 sessions of mobilization and early intervention, and orientations were given to the mother.

No previous study proposed treatment for preterm infants with DDH. The present study employed therapeutic mobilization techniques that had not been tested specifically in DDH but had shown positive results to improve joint stability in other pathologies. The protocol was based on proprioceptive neuromuscular facilitation (PNF) [26, 27].

Joint approximation maneuvers (PNFs) were used to stimulate joint receptors, facilitate alpha motor neurons, and promote muscle contraction in order to improve stability [27]. Joint approximation also offered reaction forces that may have optimized joint stability [32, 33]. The protocol was based on PNF and involved mobilization with hip adduction, flexion, and external rotation and joint approximation. Instructing the mother to perform approximation maneuvers for ten minutes (five minutes each leg), four times a day, and correct positioning of the infant helped improve hips stability because they assured a much higher amount of stimuli.

Early intervention reduces the risk of the adverse developmental outcomes related to preterm birth. However, preterm infants must be followed up and handled with caution because they are vulnerable to breathing and feeding difficulties and they have a high risk of infection and hypothermia [34]. Recent studies observed a higher frequency of alert states and orally directed behaviors after multisensory stimulation. Therefore, auditory, visual, tactile, and vestibular stimulations may have helped behavioral organization and weight gain [30, 31] and also optimized the outcome.

The protocol applied in the present study resulted in improved hip stability at 40 5/7 weeks and negative Barlow and Ortolani tests after ten sessions of mobilization and early intervention, during hospital stay. In conclusion, this case report points out that a protocol with approximation maneuvers performed with the hip with external rotation, abduction, and flexion and early intervention based on sensorimotor stimulation can be part of DDH treatment in infants at preterm age.

## Data Availability

Information about the patient was collected from the chart in Hospital Universitário da Universidade de São Paulo.
